# P-1273. Connecting Underserved Communities With Saliva-Based PCR Testing for Respiratory Infection: Insights From A Mobile Lab-In-A-Van Pilot

**DOI:** 10.1093/ofid/ofae631.1454

**Published:** 2025-01-29

**Authors:** Brittany Choate, Ruhani Sardana, Katherine Fajardo, Anne L Wyllie, Angelique W Levi

**Affiliations:** SalivaDirect Inc, New Haven, Connecticut; Yale School of Medicine, new haven, Connecticut; Yale University, New Haven, Connecticut; Yale School of Public Health, New Haven, Connecticut; Yale University School of Medicine, New Haven, Connecticut

## Abstract

**Background:**

Partnering with community leaders, we sought to address ongoing diagnostic testing needs in underserved neighborhoods and evaluate whether a saliva-based mobile testing program could help overcome barriers to testing for uninsured and low-income individuals. This is critical as many lack a primary care provider, cannot access reliable health information, or have limited financial resources.

**Figure 3.**
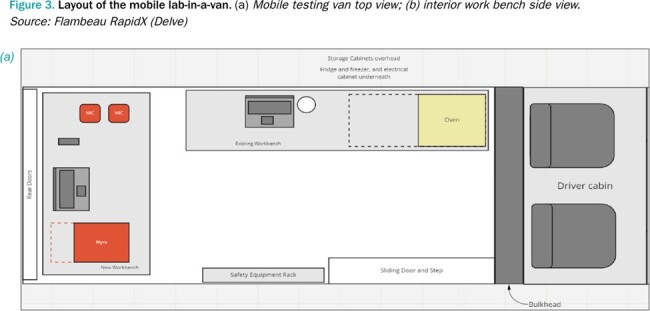
Layout of the mobile lab-in-a-van. (a) Mobile testing van top view; (b) interior work bench side view. Source: Flambeau RapidX (Delve)

**Methods:**

Free SARS-CoV-2 testing was offered at popup events using the SalivaDirect extraction-free RT-qPCR protocol on a CLIA licensed van operated by Yale Pathology Labs under FDA Emergency Use Authorization; diagnostic results were available in as little as two hours. Testing locations were identified and advertised in partnership with the community. Patient perspectives on approachability, convenience, and usefulness of mobile testing were recorded via REDcap.

**Figure 7.**
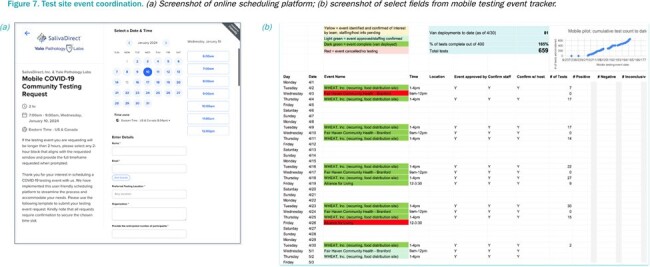
Test site event coordination. (a) Screenshot of online scheduling platform; (b) screenshot of select fields from mobile testing event tracker.

**Results:**

Between October 2023 and April 2024, 70 community events were hosted and 533 SARS-CoV-2 PCR tests administered. Across participants of all ages (0-91y, mean=42y), 79% (410/517) agreed it was easy to access the van and get a COVID test and 79% (411/517) agreed they were comfortable using the van. Of those tested, 59% (169/288) reported an annual household income of < $25,000; 92% (265/288) reported an income of < $50,000. In addition, 31% (160/517) were uninsured. Of those insured, 62% (142/228) identified “Public (Medicare, Medicaid, Tricare)” as their primary healthcare plan. Overall, 44% (128/290) reported getting the healthcare they need was a challenge, with 46% (134/289) reporting transportation challenges.

**Figure 5.**
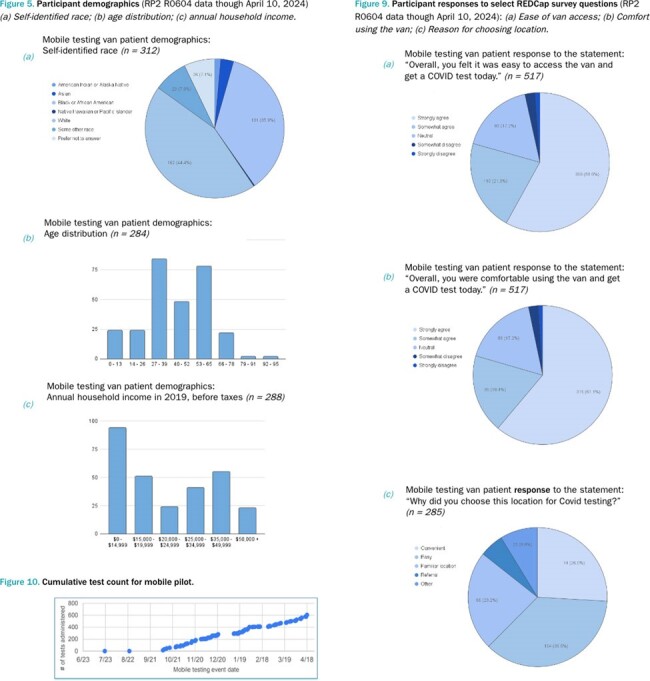
Participant demographics (RP2 R0604 data though April 10, 2024) / Figure 9. Participant responses to select REDCap survey questions (RP2 R0604 data though April 10, 2024) Figure 5. (a) Self-identified race; (b) age distribution; (c) annual household income. Figure 9. (a) Ease of van access; (b) Comfort using the van; (c) Reason for choosing location.

**Conclusion:**

This pilot demonstrated the positive impact a mobile testing program can have in addressing community needs. Analysis of participant demographics and identified barriers to service access underscores the program's effectiveness in addressing previously unmet testing requirements and enhancing healthcare accessibility within the target community. Moreover, feedback gathered from participants of all age groups via surveys reveals a strong consensus regarding the convenience and comfort of saliva-based testing at mobile events. This underscores the viability of mobile initiatives as a feasible approach to fulfilling future testing demands, particularly within underserved populations.

**Disclosures:**

**Anne L. Wyllie, PhD**, SalivaDirect: Board Member|SalivaDirect: Honoraria

